# Resistance prediction in high‐grade serous ovarian carcinoma with neoadjuvant chemotherapy using data‐independent acquisition proteomics and an ovary‐specific spectral library

**DOI:** 10.1002/1878-0261.13410

**Published:** 2023-03-19

**Authors:** Liujia Qian, Jianqing Zhu, Zhangzhi Xue, Tingting Gong, Nan Xiang, Liang Yue, Xue Cai, Wangang Gong, Junjian Wang, Rui Sun, Wenhao Jiang, Weigang Ge, He Wang, Zhiguo Zheng, Qijun Wu, Yi Zhu, Tiannan Guo

**Affiliations:** ^1^ School of Medicine Zhejiang University Hangzhou China; ^2^ Key Laboratory of Structural Biology of Zhejiang Province, School of Life Sciences Westlake University Hangzhou China; ^3^ Institute of Basic Medical Sciences Westlake Institute for Advanced Study Hangzhou China; ^4^ The Cancer Hospital of the University of Chinese Academy of Sciences (Zhejiang Cancer Hospital) Hangzhou China; ^5^ Institute of Basic Medicine and Cancer (IBMC) Chinese Academy of Sciences Hangzhou China; ^6^ Department of Obstetrics and Gynecology Shengjing Hospital of China Medical University Shenyang China; ^7^ Westlake Omics (Hangzhou) Biotechnology Co., Ltd. China; ^8^ Department of Clinical Epidemiology, Department of Obstetrics and Gynecology Shengjing Hospital of China Medical University Shenyang China

**Keywords:** chemotherapy resistance, data‐independent acquisition, machine learning, MS spectral library, ovarian cancer, targeted proteomics

## Abstract

High‐grade serous ovarian carcinoma (HGSOC) is the most common subtype of ovarian cancer with 5‐year survival rates below 40%. Neoadjuvant chemotherapy (NACT) followed by interval debulking surgery (IDS) is recommended for patients with advanced‐stage HGSOC unsuitable for primary debulking surgery (PDS). However, about 40% of patients receiving this treatment exhibited chemoresistance of uncertain molecular mechanisms and predictability. Here, we built a high‐quality ovary‐specific spectral library containing 130 735 peptides and 10 696 proteins on Orbitrap instruments. Compared to a published DIA pan‐human spectral library (DPHL), this spectral library provides 10% more ovary‐specific and 3% more ovary‐enriched proteins. This library was then applied to analyze data‐independent acquisition (DIA) data of tissue samples from an HGSOC cohort treated with NACT, leading to 10 070 quantified proteins, which is 9.73% more than that with DPHL. We further established a six‐protein classifier by parallel reaction monitoring (PRM) to effectively predict the resistance to additional chemotherapy after IDS (Log‐rank test, *P* = 0.002). The classifier was validated with 57 patients from an independent clinical center (*P* = 0.014). Thus, we have developed an ovary‐specific spectral library for targeted proteome analysis, and propose a six‐protein classifier that could potentially predict chemoresistance in HGSOC patients after NACT‐IDS treatment.

## Graphical abstract

About 40% of the patients receiving neoadjuvant chemotherapy exhibit chemoresistance of uncertain molecular mechanisms and predictability. Using global proteomics and machine learning, 40 proteins were found associated with chemoresistance. After verification by targeted proteome, a six‐protein classifier was built to effectively predict the resistance to additional chemotherapy, which were validated in an independent cohort.

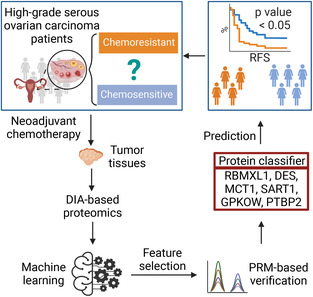

**Abbreviations**
AUC: area under curve
CiRT: common internal retention time
CKB: creatine kinase B‐type
DDA: data‐dependent acquisition
DES: desmin
DIA: data‐independent acquisition
DPHL: DIA pan‐human spectral library
EOC: epithelial ovarian cancer
FFPE: formalin‐fixed and paraffin‐embedded
HCD: higher energy collisional dissociation
HDAC8: histone deacetylase 8
HGSOC: high‐grade serous ovarian carcinoma
IDS: interval debulking surgery
MCT1: monocarboxylate transporter 1
NACT: neoadjuvant chemotherapy
OCT: optimal cutting temperature
OVLib: ovary spectral library
PCA: principal component analysis
PCT: pressure cycling technique
PDS: primary debulking surgery
PHL: pan‐human library
PRM: parallel reaction monitoring
PTBP2: polypyrimidine tract‐binding protein 2
QE‐HF: Q exactive HF hybrid quadrupole‐orbitrap
RFS: relapse‐free survival
RT: retention time
SRM: selected reaction monitoring

## 1 Introduction

Ovarian cancer is the ninth most common cancer among females worldwide [1] and the most fatal tumor of the female reproductive system in the United States [2]. For patients with advanced‐stage ovarian cancers who are not suitable candidates for primary debulking surgery (PDS), management with neoadjuvant chemotherapy (NACT) and interval debulking surgery (IDS) is a potentially beneficial option as reducing residual disease could improve surgical outcomes [3]. However, compared with advanced‐stage patients receiving PDS, no significant survival benefit was observed in patients receiving NACT‐IDS [4, 5, 6, 7]. It has been reported that 44.2% of patients treated with NACT‐IDS were chemoresistant, which is higher than those treated with PDS (31.2%) [8]. Thus, the NACT‐IDS treatment‐induced platinum resistance may be one among several causes for this unfavorable treatment response. Both the reduction of CA125 levels [9] and sequential F‐18‐fluorodeoxyglucose positron emission tomography [10] during NACT have been reported to be associated with cytoreduction outcomes at IDS and long‐term prognosis. Higher levels of stromal tumor‐infiltrating lymphocytes both pre‐ and post‐NACT [11] and reduced expression of certain homologous recombination genes [12], as well as the decreased expression of the stemness marker, ALDH1 [13], have been found associated with favorable outcomes in patients with NACT by Cox proportional hazard regression models. Lee et al. [14] performed multiomics profiling, including proteomics, of HGSOC samples from patients treated with complete PDS or NACT, and cataloged multiple altered molecules and pathways among groups with poor or favorable outcomes.

Recently, advanced proteomic technologies applied in primary ovarian cancers have reported (a) biomarkers for differential diagnosis of histotypes [15, 16]; (b) patient stratification for precision therapy [17, 18, 19, 20, 21]; and (c) potential therapeutic targets [22, 23, 24]. However, no study has yet systematically characterized protein dysregulations which underpin chemotherapy resistance after NACT‐IDS or provided a predictive model for resistance and sensitivity to chemotherapy after NACT‐IDS treatment.

DIA mass‐spectrometry (DIA‐MS)‐based quantitative proteomics enables comprehensive and permanent digital profiling of LC–MS‐compatible peptide precursors from clinical specimens with high reproducibility and throughput [25, 26, 27]. Thus, it has found to be increasingly used in clinical applications to identify dysregulated proteins in disease states. Although several library‐free tools for untargeted analysis of DIA have been developed, library‐query targeted approaches for interpreting DIA data are still a widely used strategy, owing to its high specificity for detecting proteins expressed in particular tissue types [27, 28, 29].

Targeted proteomics, such as selected reaction monitoring (SRM) [30] and PRM [31], has emerged to verify and validate expression of selected proteins in complex proteomes, allowing reproducible measurement of up to about 100 proteins of interest in a single analysis. Spectral libraries are required for developing SRM or PRM assays in targeted proteomics.

Several prebuilt pan‐human spectral libraries using both TripleTOF (pan‐human library, PHL) [32] and Orbitrap (DIA pan‐human library, DPHL) [33] spectral data and an ovary‐specific library using TripleTOF spectral data [34] have been published. PHL and DPHL contain spectral information of over 10 000 unique proteins. These pan‐human libraries, although comprehensive, are inferior to tissue‐specific libraries [35] mainly because of false negatives resulting from larger search space and technical variability of instruments in the case of published human spectral libraries. Optimizing the spectral library size into a subset library from a prebuilt pan spectral library helps improve proteome coverage by DIA [36]. Of note, both PHL and DPHL do not contain spectral data for ovarian tissue specimens [32, 33]. To date, only one ovary‐specific library using peptides from primary HGSOC and Orbitrap spectral data has been published but contains fewer than 8000 proteins [37]. Thus, there is need for an in‐depth tissue‐specific library of ovarian tissue specimens representing different histopathological diagnoses to expand the spectral library for interrogating the ovarian tissue proteome.

In this study, we developed a comprehensive spectral library for ovarian tissue specimens and applied it to propose a protein‐based classifier for predicting chemoresistance in HGSOC patients after NACT‐IDS treatment.

## 2 Materials and methods

### 2.1 Patients and samples

This study was approved by the Medical Ethics Committees of the Cancer Hospital of the University of Chinese Academy of Sciences (IRB‐2020‐155), Shengjing Hospital of China Medical University (2015PS28K), and Westlake University (20190401GTN0009, 20221124GTN003). The study methodologies followed the standards set by the Declaration of Helsinki, and the experiments were undertaken with the understanding and written consent of each subject.

To generate an ovary‐specific spectral library, 167 surgically resected ovarian tissues, including 33 cases of normal tissues from patients with uterine myoma or cervical cancer without histologically documented ovarian involvement, 44 cases of benign tissues, 10 cases of borderline tissues, 35 cases of epithelial ovarian cancer (EOC) tissues obtained from PDS, 20 cases of EOC tissues from relapsed patients, and 25 cases of primary EOC tissues with NACT [38] were collected from the Cancer Hospital of the University of Chinese Academy of Sciences between 2006 and 2018. Details of the histopathology of borderline tumors are provided in Table S1. Nine of 10 borderline tumors were serous, while one of them was mucinous. Twenty‐eight of 35 ovarian carcinomas dissected by PDS were HGSOC, while only one was a low‐grade serous carcinoma. Among 35 primary EOC tissues by PDS, we also included one mucinous adenocarcinoma, three endometrioid carcinomas, and two clear cell carcinomas. The proportions of these histological types were similar to their natural incidence rates. Detailed sample and patient information are provided in Table S1.

Seventy‐one ovarian cancer tissue samples were collected from 63 patients treated with NACT‐IDS in the Cancer Hospital of the University of Chinese Academy of Sciences (cohort A) between 2009 and 2017. All patients had late‐stage HGSOC and all had received two or three cycles of platinum‐based neoadjuvant therapy and at least six cycles of chemotherapy in total. For the purpose of machining learning, this NACT‐IDS cohort was divided into training (*N* = 36, *n* = 42) and test (*N* = 27, *n* = 29) subcohorts by year of diagnosis (where *N* denotes the number of patients, and *n* denotes the number of specimens). For external validation, 62 ovarian cancer tissue samples of 57 HGSOC patients treated with NACT‐IDS were collected from Shengjing Hospital of China Medical University (cohort B) between 2013 and 2019. Detailed patient information is listed in Table S1. Patients who relapsed within 6 months after the last cycle of adjuvant therapy were considered to be the resistant group, while those who relapsed after 6 months since the last cycle of adjuvant therapy were grouped as the sensitive group. Specimens of cohort A were embedded in optimal cutting temperature (OCT) compound, while specimens of cohort B were formalin fixed and paraffin embedded (FFPE). Histological features and proportions of tumor nuclei were evaluated in histological sections stained with hematoxylin and eosin. All tumor samples contained at least 60% tumor nuclei.

### 2.2 Protein extraction and digestion assisted by pressure cycling technique

To remove OCT, each fresh frozen tissue sample (~ 1 mg) was washed in sequential concentrations (volume/volume) of ethanol‐water as follows: 70% (30 s), 0% (30 s), 70% (5 min, twice), 85% (5 min, twice), and 100% (5 min, twice) [39]. FFPE tissue samples were firstly dewaxed using heptane, rehydrated using sequential concentrations (volume/volume) of ethanol‐water (100%, 90% and 75%), hydrolyzed in 0.1% formic acid and finally in 100 mm Tris–HCl (pH 10) [40]. Thereafter, tissue samples were transferred into pressure cycling technique (PCT) tubes and processed into peptide samples by PCT‐assisted tissue lysis and protein digestion according to the published protocol [40].

### 2.3 Spectral library generation

Peptide samples from four groups of tissue specimens were pooled, respectively, for the spectral library building. These four groups were normal (*N* = 33), benign (*N* = 44), primary malignant samples obtained from PDS (*N* = 45; 10 borderline cases and 35 carcinoma cases), and postchemotherapy samples (*N* = 45; 20 cases from relapsing EOC cohort and 25 cases from EOC cohort treated with NACT). Using Thermo Ultimate Dionex 3000 (Thermo Fisher Scientific, San Jose, CA, USA), each pooled peptide sample was fractionated into 60 aliquots as previously described [41]. The 60 aliquots of three pooled peptides of normal, benign and primary malignant samples from PDS were combined into 10 fractions in the following combination scheme: 1 + 11 + 21 + 31 + 41 + 51, 2 + 12 + 22 + 32 + 42 + 52, …, 10 + 20 + 30 + 40 + 50 + 60 (Table S1). Similarly, pooled peptide fractions of postchemotherapy samples were combined into 15 fractions in the following scheme: 1 + 16 + 31 + 46, 2 + 17 + 32 + 47, …, 15 + 30 + 45 + 60 (Table S1). Each fraction was dried and then redissolved in buffer A (2% ACN, 0.1% formic acid), before further separation in a 60‐min gradient and analyzed by Q Exactive HF hybrid Quadrupole‐Orbitrap (QE‐HF; Thermo Fisher Scientific) in data‐dependent acquisition (DDA) mode with the same settings as described previously [42]. The top 20 precursors were fragmented using higher energy collisional dissociation (HCD). Eighteen fractions randomly selected from the 45 fractions were injected twice as technical replicates. Additional details are provided in Table S1. In total, 63 DDA files were generated and analyzed by Spectronaut (Version 13.5.190902.43655, Biognosis, Schlieren, Switzerland) against a Swiss‐Prot human protein database (downloaded on 9 February 2018) comprising 20 555 reviewed proteins. All parameters were by default according to the BGS Factory Settings in Spectronaut. The ovarian spectral library is hereafter referred to as OVLib.

### 2.4 PulseDIA and DIA data analysis

Two hundred and fifty nanogram of peptide samples were separated over a 30‐min LC gradient on a nanoflow Dionex UltiMate 3000 RSLCnano System and then analyzed by a QE‐HF with the PulseDIA in two parts as described previously [43]. Five peptide samples were randomly selected as technical replicates and MS data acquisition was performed twice for them.

The DIA raw data were firstly converted into mzML format filtered by peakPicking by msconvert (v3.0), and then analyzed by dia‐nn (v1.8.1) against the DPHL [33] and the OVLib, respectively. All parameters of dia‐nn were set by default.

### 2.5 Quality control and statistical analysis

Pearson correlation was calculated by log_2_(intensity) of protein abundance between replicates. Intensities from technical replicates were averaged. Proteins with over 70% missing values in the sample set were filtered out, after which missing values of the remaining proteins were imputed as 0.8*minimum. Unpaired Student's *t* test by log_2_(intensity) was performed between sensitive and resistant groups of the training cohort. Fold change was calculated from the means of protein intensities between these two groups. Criteria for differentially expressed proteins were that the *P* < 0.05 and fold change > 2. Statistical analysis was performed by r (version 4.0.5). Pathway enrichment for differentially expressed proteins was performed by metascape (v3.5).

### 2.6 Machine learning

Criteria for selecting differential features between the sensitive and resistant groups of the training cohort by Student's *t* test were *P* value < 0.05 and fold change > 1.5. The protein matrix of 145 features by DIA was normalized using Z‐score, and the same Z‐score normalization was applied to the test cohort. Fifty‐four proteins in the training set with mean decrease accuracy larger than 1.5 were first selected using the r package randomForest (version 4.6.14). Then, we randomly split the training set into 80% of samples (*n* = 34) for training to build 1000 trees and remaining 20% samples (*n* = 8) for internal validation by the hold‐out method. This process was repeated 250 times. We selected the model with highest accuracy rate in validation set and evaluate its prediction utility in an independent test (*n* = 29) cohort.

### 2.7 Targeted proteome by PRM

Firstly, the expression of 30 out of 40 proteins from the prognosis model was verified by PRM. For retention time calibration, 15 peptides were selected from OVLib as common internal retention time (CiRT) standard peptides following the procedures described previously [33] (Table S2). The peptides were separated at 300 nL·min^−1^ over a 45‐min LC gradient from 5% to 30% buffer B (buffer A: 2% ACN, 0.1% formic acid; buffer B: 98% ACN, 0.1% formic acid) in UltiMate™ 3000 RSLCnano System (Thermo Fisher Scientific). The ionized peptides were transferred into QE‐HF. Fifty‐four peptides (including 15 CiRT peptides, Table S2) were selected and analyzed in a ± 3 min time window by time‐scheduled acquisition. The full scans were performed at a resolution of 60 000 and *m/z* from 400 to 2000 were collected. The AGC target was set as 3e6 with maximum IT at 55 ms. The isolation window for target precursors was set as 1.6 with normalized collision energy at 27%. The product ions were collected at a resolution of 30 000, AGC target of 2e5 and maximum injection time of 80 ms.

Next, we established a short‐gradient PRM method for quantification of six proteins in the final model to analyze the selected proteins in the independent validation cohort. The peptides were separated over a 15‐min LC gradient from 10% to 42% buffer B in UltiMate™ 3000 RSLCnano System (Thermo Fisher Scientific). Twenty‐three peptides (including 15 CiRT peptides, Table S2) were selected and analyzed in a ± 2.5‐min time window by time‐scheduled acquisition. Other parameters were identical as that in the 45‐min gradient PRM methods.

## 3 Results and discussion

### 3.1 Ovarian tissue spectral library

**Fig. 1:**
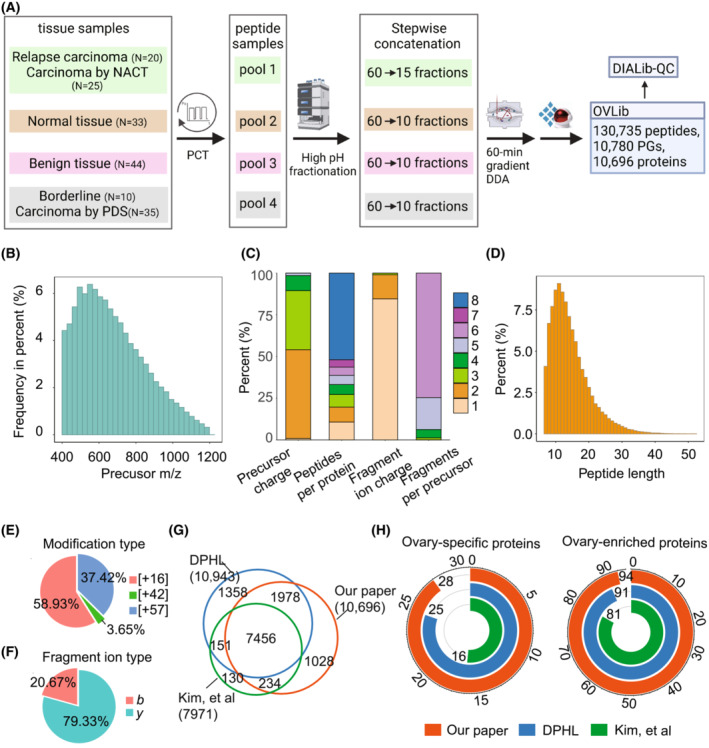
The ovary spectral library. (A) Workflow for the generation of an OVLib. PGs represent protein groups. (B) The distribution of precursor *m/z*. NACT, Neoadjuvant chemotherapy; PDS, Primary debulking surgery; PCT, Pressure cycling technique; DDA, data‐dependent acquisition; OVLib, Ovary spectral library; DIALib‐QC, DIA library quality control; PGs, protein groups. (C) The distribution of precursor charge states, proteotypic peptides for each protein, fragment ions per precursor ion, and fragment ion charge states. *m/z*, Mass‐to‐charge ratio. (D) The distribution of peptide lengths. (E) The distribution of three modifications (16: oxidation in methionine, 42: acetylation in N terminal, 47: carbamidomethylation in cysteine). (F) The proportion of *b*, *y* ions. (G) The Venn plot of unique proteins among OVLib and two prepublished libraries. DPHL, DIA pan‐human spectral library. The counts of ovary‐specific proteins and ovary‐enriched proteins (H) among OVLib and two prepublished libraries.

To generate the OVLib, we firstly processed 167 surgically resected ovarian tissues consisting of 33 cases of normal tissues, 44 cases of benign tissues, 10 cases of borderline tissues, 35 cases of EOC tissues by PDS, 20 cases of EOC tissues from relapsed patients, and 25 cases of EOC tissues with NACT (Fig. 1A). Peptides from these fresh frozen tissues were prepared using PCT [26, 40, 44]. Peptides from normal tissues (*n* = 33), benign tissues (*n* = 44), malignant tissues by PDS (*n* = 45) and postchemotherapy tissues (*n* = 45) were combined into four pooled samples (Fig. 1A). Ten to 15 fractions of each pooled sample were separated by high pH fractionation, and 63 injections (including 18 technical replicates) were acquired using 60‐min gradient DDA on Orbitrap MS instruments (Fig. 1A). The OVLib built by Spectronaut contained 175 769 precursors, 130 735 proteotypic peptides, 10 780 protein groups, and 10 696 unique proteins (Fig. 1A). The proteomic depth achieved here is higher than those reported in the literature [21, 22].

We next assessed the complexity and characteristics of OVLib by DIAlib‐QC [45]. The precursors ranged from 400 to 1200 *m/z* primarily ionized at two (53.24%) or three (35.35%) charges (Fig. 1B,C). The retention time (RT) between [M + 2H]^2+^ and [M + 3H]^3+^ charge states of the same peptide was calibrated using Biognosys's Deep Learning Assisted iRT Calibration. High RT correlation has been reported to improve selectivity of chromatogram extraction for DIA analysis, leading to increased identifications compared to other studies [45]. The range of peptide length covered was from 7 to 52, and 82.52% of them were between 8 and 20 (Fig. 1D). Oxidation was the most common modification and detected in 9509 peptide precursors (Fig. 1E). Most (89.27%) of proteins were identified from at least two peptides, and up to 5647 proteins were identified from more than seven peptides (Fig. 1C). Over 93% of precursors generated at least five fragments (Fig. 1C), and significantly more fragments with y ion (79.33%) were detected than those with b ions (20.67%) (Fig. 1F). Most fragment ions were observed in charge one (84.53%) and two (14.42%) states (Fig. 1C). The overall characteristics of our spectral library are consistent with previous reports of pan‐human proteome libraries [32, 33].

We compared the OVLib with the two published libraries, namely the DPHL [33] and the prebuilt ovary‐specific library based on Orbitrap [37]. The HCD protein numbers identified in the OVLib were comparable with those in DPHL and more than 90% of identified proteins overlapped in both libraries, while unique proteins in the prebuilt ovary‐specific library were significantly fewer than those in OVLib (Fig. 1G). Tissue‐specific and ‐enriched proteins have been reported to mediate physiological functions of the tissue [46]. In the OVLib, 28 out of 31 annotated ovary‐specific proteins and 94 out of 97 annotated ovary‐enriched proteins were identified [46], which exceed those identified in both two published libraries (Fig. 1H). This demonstrates the advantages of OVLib for characterizing ovarian tissue‐specific proteins more comprehensively.

### 3.2 Proteomic analysis of HGSOC samples from patients with NACT‐IDS

**Fig. 2:**
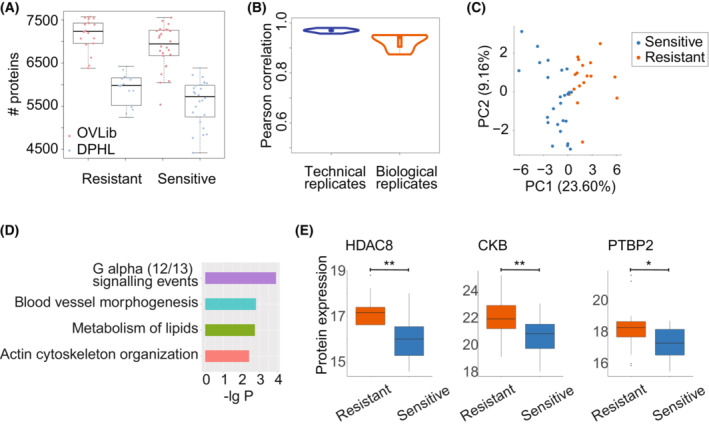
Quality control of the proteome against OVLib and its application. (A) The comparison of the unique proteins identified in ovarian cancer tissues against DPHL and OVLib. The plot whiskers define outliers according to Tukey's rule (e.g., more than 1.5 times the interquartile range from the quartiles). (B) The Pearson correlation of the quantified proteins between technical replicates and biological replicates. The biological replicates represent the proteomic data obtained from different ovarian tissue samples which are dissected from the same patients, while the technical replicates represent the proteomic data that was run twice using the same peptide samples. Eight pairs of biological replicates and five pairs of technical replicates were included. (C) Unsupervised clustering by differentially expressed proteins using PCA. (D) The enriched pathways for differentially expressed proteins by metascape. (E) The protein expression of differentially expressed proteins between the sensitive and resistant groups. The *P* values were calculated by Student's *t* test. **, *P* value < 0.01; *, 0.01 ≤ *P* value < 0.05. The plot whiskers define outliers according to Tukey's rule.

We next applied OVLib to study the proteome of ovarian cancer tissues of patients treated with NACT‐IDS. We profiled the proteome of 71 ovarian tumor samples from 63 cases of patients using the PulseDIA method [43] (Table S2). The DIA data were analyzed against both DPHL and our OVLib using dia‐nn (v1.8) with the same setting. A total of 114 754 peptides and 10 070 proteins were identified against the OVLib compared with 107 132 peptides and 9177 proteins by DPHL (Fig. 2A, Table S2). 7.11% more peptides and 9.73% more proteins were identified by the OVLib, compared with those by the DPHL. In addition, 13.42% and 7.17% fewer missing values were observed in the peptide and protein matrices, respectively (Table S2). Pearson correlations of protein quantification were 97.0% for the technical replicates and 94.2% for the biological replicates (Fig. 2B), indicating a high degree of reproducibility of DIA‐MS data.

### 3.3 Protein classifier to predict chemotherapy resistance

**Fig. 3:**
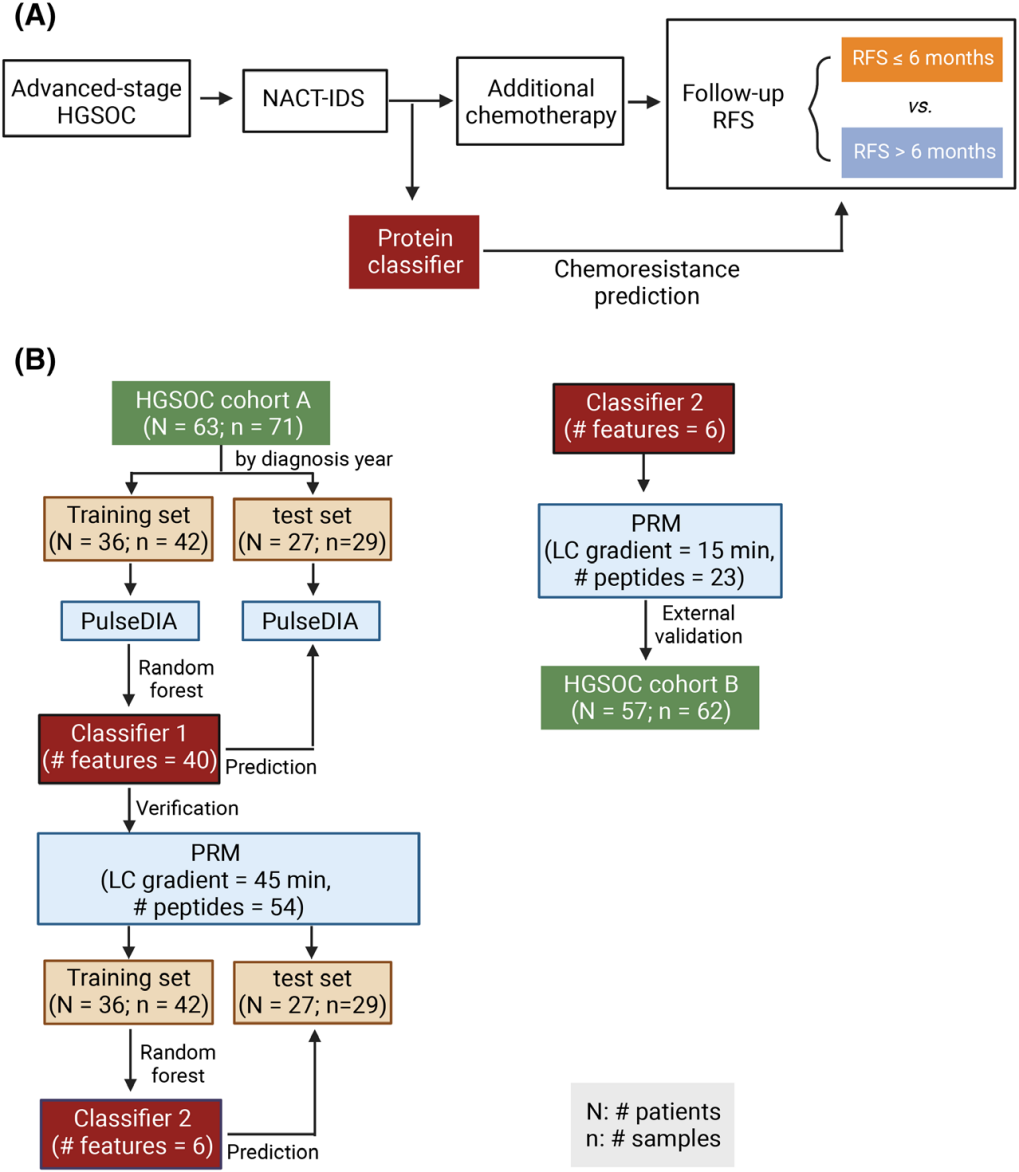
The schematic study design to establish a protein classifier for predicting chemoresistance of HGSOC patients after NACT‐IDS treatment. (A) The rationale for the protein classifier. (B) The workflow of establishing the protein classifier.

We next divided these patients into a training cohort (*n* = 42) and a test cohort (*n* = 29) by the year of diagnosis (Table S1). A total of 40 proteins exhibited differential expression between sensitive (*n* = 25) and resistant (*n* = 17) ovarian cancer tissue samples of the training cohort (Table S3, *P* value < 0.05 and fold change > 2). These differentially expressed proteins, which clearly separated the resistant and sensitive groups in principal component analysis (PCA; Fig. 2C), were enriched in G alpha (12/13) signaling events, blood vessel morphogenesis, metabolism of lipids, and actin cytoskeleton organization (Fig. 2D). Among them, several proteins have been reported and validated to be associated with tumorigenesis and resistance in ovarian cancer. For example, our data showed significant upregulation of histone deacetylase 8 (HDAC8), creatine kinase B‐type (CKB), and polypyrimidine tract‐binding protein 2 (PTBP2) in the resistant group (Fig. 3E). HDAC enzymes deacetylate histones and modulate the transcription of multiple tumor suppressor genes [47]. In addition, HDAC inhibitors have known anticancer activities and are synergistic in clinical chemotherapeutics not only in ovarian cancer and multidrug‐resistant cell lines but also in xenografts [48, 49]. Creatine kinase B‐type participates in energy homeostasis by reversibly transferring phosphate between ATP and phosphogens. CKB knockout in an ovarian cancer cell line induced G2 arrest, sensitivity to chemotherapeutic agents, and a tumor‐suppressive metabolic state of decreased glycolysis but increased oxidative phosphorylation [50]. Protein expression of PTBP was upregulated in the ovarian cancers compared to normal tissues, and its knockdown inhibited tumor cell proliferation and invasiveness [51], possibly due to the effect of PTBP knockdown on reducing alterative splicing of multidrug resistance protein [52].

**Fig. 4:**
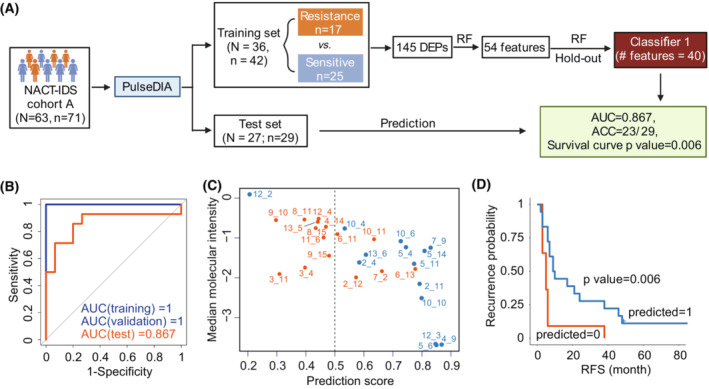
Protein classifier for predicting chemoresistance of HGSOC patients after NACT‐IDS treatment. (A) Development of a 40‐protein classifier using random forest based on PulseDIA data. RF, random forest analysis. (B) The receiver operating characteristic curves (ROCs) of training, validation set and test cohorts by the 40‐protein classifier. (C) Classification of tumors by the 40‐protein classifier in the test cohort. The orange ones were predicted chemoresistant, while the blue ones chemosensitive. (D) The Kaplan–Meier plot of the predicted groups by the 40‐protein classifier in the test cohort. The *P* was calculated by Log‐rank test.

To distinguish patients resistant to platinum‐based additional chemotherapy from the NACT‐IDS cohort (Fig. 3A), we performed random forest analysis using dysregulated proteins between resistant and sensitive groups (Figs 3B and 4A, Table S3). Forty proteins were prioritized by the ranked order of importance by random forest analysis (Table S3). The area under the curve (AUC) for internal validation dataset reached 1 (Fig. 4B). In the test dataset, this model correctly identified 23 out of 29 patients, achieving an AUC of 0.867 (Fig. 4B,C). The two groups predicted by this model observed significant differences in relapse‐free survival (RFS) (Log‐rank test, *P* value = 0.006; Fig. 4D). Computational procedures are detailed in the Section 2.

### 3.4 Verification of the protein classifier by PRM

**Fig. 5:**
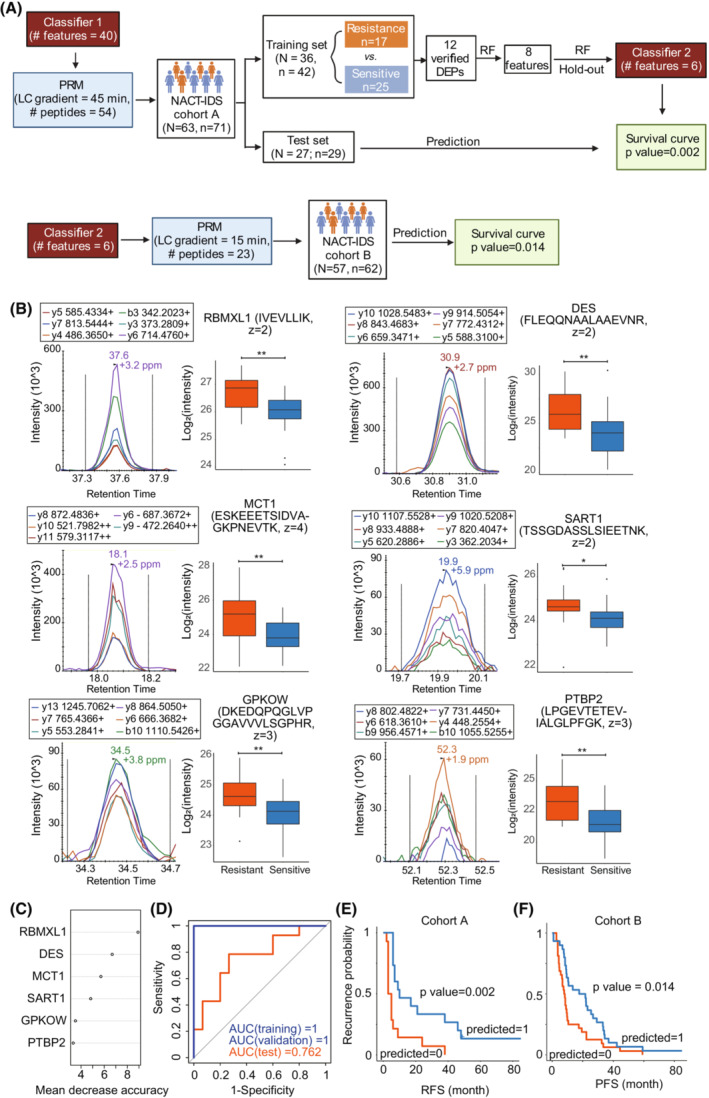
Six‐protein classifier for predicting chemoresistance of HGSOC patients after NACT‐IDS treatment. (A) Development of a six‐protein classifier using random forest based on PRM data. RF, random forest analysis. (B) The representative peak group chromatography (left) of top six features selected by RF analysis, and their expression between the resistance and sensitive groups in the training set. The *P* was calculated by Student's *t* test. **, *P* < 0.01; *, 0.01 ≤ *P* < 0.05. z represents the charge state of a peptide precursor. The plot whiskers define outliers according to Tukey's rule. (C) The rank of mean decrease accuracy of the six features. (D) The ROCs of training, validation set and test cohort by the six‐protein classifier. (E) The Kaplan–Meier plot of the predicted groups by the six‐protein classifier in the test cohort. The *P* was calculated by Log‐rank test. (F) The Kaplan–Meier plot of the predicted groups by the six‐protein classifier in the external validation cohort. The *P* was calculated by Log‐rank test.

To evaluate and verify these protein features in a more sensitive, reproducible and high‐throughput assay for potential clinical application, we performed PRM quantification of these proteins in the prognosis model (Fig. 3B), and established robust assays for 39 peptide precursors from 30 proteins (Table S2). Among them, 12 proteins were verified to be dysregulated between resistant and sensitive groups of training cohort (Table S3). Eight out of 12 proteins with mean decrease accuracy larger than three were selected by random forest (Fig. 5A). After hold‐out validation, a six‐protein model (RBMXL1, DES, MCT1, SART1, GPKOW, and PTBP2) was established (Fig. 5B,C). Desmin (DES) is a marker for tumor‐associated fibroblasts (TAFs), indicating myofibroblast and provascularizing potential. The overexpression of monocarboxylate transporter 1 (MCT1) has been reported to be correlated with cisplatin resistance in both ovarian tumor tissues and cell lines [53]. In addition, its knockdown in both cell lines and xenograft model reversed cisplatin resistance and activated Fas/FasL pathway [53]. Using this prognosis model, AUCs for internal validation and test cohort were 1 and 0.762, respectively (Fig. 5D). In the test cohort, 22 out of 29 patients were correctly identified; of the seven misclassified patients, four relapsed in 6 months after the last chemotherapy (Table S3), which is the defining point of RFS for chemoresistance. Similarly, the RFS of the two groups predicted by this model showed a significant difference (Log‐rank test, *P* value = 0.002, Fig. 5E). To further determine the validity of this prognostic model, we applied it to a validation cohort from an independent clinical center (cohort B; Fig. 3B) and found significant differences of progression‐free survival (Log‐rank test, *P* value = 0.014) between the two predicted groups (Fig. 5F).

## 4 Conclusions

In summary, we present an ovary‐specific spectral library for targeted proteome analysis of ovarian tissues. We propose here a six‐protein classifier to distinguish the resistant and sensitive groups of HGSOC patients after NACT‐IDS treatment. This six‐protein classifier is based on PRM‐MS and could be potentially applied in clinical management. Further trials of this classifier in multicenter prospective HGSOC cohorts should be carried out in the future to investigate its potential utility in the clinical management of patients with ovarian cancers.

## Conflict of interest

TGu and YZ are shareholders of Westlake Omics Inc. WGe is an employee of Westlake Omics Inc. The other authors declare no conflict of interest.

## Author contributions

TGu, LQ, YZ, and RS designed the project. ZZ, JZ, WGo, TGo, QW, and JW collected samples. LQ, NX, LY, and XC performed the proteomic experiments. LQ, ZX, HW, WJ, and WGe conducted proteomic data analysis. LQ wrote the manuscript with inputs from all co‐authors. TGu, YZ, and RS revised the manuscript. TGu, YZ, QW, and ZZ supervised the project.

### Peer review

The peer review history for this article is available at https://publons.com/publon/10.1002/1878‐0261.13410.

## Supporting information


**Table S1.** Clinical characteristics of all patients.

**Table S2.** Peptide and protein matrices.

**Table S3.** Differentially expressed proteins and the results of machine learning.

**Data S1.** Legends.

## Data Availability

The mass spectrometry proteomics data have been deposited to the ProteomeXchange Consortium (http://proteomecentral.proteomexchange.org) via the iProX partner repository [54, 55] with the dataset identifier PXD039160.
