# Supplementary material for: Resistance prediction in high‐grade serous ovarian carcinoma with neoadjuvant chemotherapy using data‐independent acquisition proteomics and an ovary‐specific spectral library
Source: Mol Oncol. 2023 Mar 19;17(8):1567–80. doi: 10.1002/1878-0261.13410 (PMC10399723; doi:10.1002/1878-0261.13410)
Supplement: Supplementary file 4 — Data S1. Legends. [file MOL2-17-1567-s002.docx]

**Supplementary Table Legend**

**Supplementary Table 1. Clinical characteristics of all patients.**

**A. Clinical information of samples for library building**

**B. Fraction information of samples for library building**

**C. Clinical information of ovarian cancer patients treated with NACT-IDS (Cohort A).** Age at diagnosis, histological type, FIGO stage, metastasis of lymph, frequency of chemotherapy, recurrence-free survival, CA125 before the treatment, HE4 before the treatment, CA125 of drug resistance before last chemotherapy, frequency of neoadjuvant therapy, and neoadjuvant therapy are listed. PTX, paclitaxel; DDP, Cisplatin; CBP, carboplatin; Pt, platinum; TNF, Tumor Necrosis Factor; DXM, dexamethasone.

**D. Sample information of ovarian cancer patients treated with NACT-IDS (Cohort A).**

**E. Sample information of ovarian cancer patients treated with NACT-IDS (Cohort B).**

**Supplementary Table 2. Peptide and protein matrices.**

**A. Peptide matrix against OVLib.**

**B. Protein matrix against OVLib.**

**C. Peptide matrix against DPHL.**

**D. Protein matrix against DPHL.**

**E. CiRT for PRM assays.**

**F. Peptide list of the 45-min PRM experiments.**

**G. Protein matrix of the 45-min PRM experiments.**

**H. Peptide list of the 15-min PRM experiments.**

**I. Protein matrix of the 15-min PRM experiments.**

**Supplementary Table 3. Differentially expressed proteins and the results of machine learning.**

**A. Differentially expressed proteins between the resistant and sensitive groups of training cohort by DIA assay.**

**B. Forty features selected by random forest analysis.**

**C. Differentially expressed proteins between the resistant and sensitive groups of training cohort by PRM.**

**D. Prediction result of test cohort by the six-protein classifier.**

**E. Prediction result of external validation cohort by the six-protein classifier.**
